# The functional regulation of protein composite nanofibers on human astrocyte for neural regeneration

**DOI:** 10.21203/rs.3.rs-8330012/v1

**Published:** 2025-12-19

**Authors:** Li Yao, Karen Bustamante-Fuchs, Kayla Cantu, Teresa Shippy

**Affiliations:** 1 Department of Biological Sciences, Wichita State University, 1845 Fairmount Street, Wichita, KS, USA 67260; 2 K-INBRE Data Science Core, Division of Biology, Kansas State University, Manhattan, KS, USA 66506

**Keywords:** Astrocyte, nanofiber, collagen, soy protein, migration, signaling pathway

## Abstract

Collagen is an extracellular matrix molecule, and soy protein can potentially modulate neural immune activity. Nanofiber scaffolds fabricated from collagen and soy protein may enhance the neural repair process by conducting neural growth and regulating the immune response of neural tissue. Studies have reported that transplantation of fetal astrocytes may stimulate axonal regeneration and functional recover after spinal cord injury. Nanofibers may act as a matrix carrier for cell transplantation to enhance cell therapy. In this study, we fabricated nanofibers by collagen, soy protein isolate (SPI), and polycaprolactone (PCL) using electrospinning. The nanofibers were characterized by scanning electron microscopy (SEM), Fourier transform infrared (FTIR) spectroscopy, and contact angle assays. Human fetal astrocytes showed high viability on the nanofibers. Flow cytometry analysis showed that the SPI component in collagen/SPI/PCL nanofibers does not affect the cell cycle compared with the collagen/PCL nanofibers. The aligned fibers showed clear guidance for cell migration along the fibers. RNA-sequencing analysis revealed that the “neurodegeneration” and “antigen processing and presentation” pathways are enriched in down-regulated genes including IL1B, IL6, HLA-B, HLA-DMB, HLA-DPA1, and HLA-DRA for the cells on collagen/SPI/PCL fibers compared with collagen/PCL fibers. The “focal adhesion” pathway is enriched in up-regulated genes including COL4A1, COL4A2, FN1, LAMB1, LAMB2, AKT2, RAC1, RAC2, ROCK2, and PIP5K1A. These results suggest that SPI may regulate the cell immune response and cell adhesion and motility in the neural repair process.

## INTRODUCTION

Neural injury such as spinal cord injury (SCI) can cause cell death of neurons and glia and the degeneration of axons. The excessive immune reaction and the reactive scar formed in the lesion results in further damage of the neural tissue [1,2]. Soy protein has been recently recognized as an attractive biomaterial to generate bioscaffold that can be applied in tissue repair [3,4] because soy isoflavones and their metabolites regulate the in vivo immunological reaction [5,6]. Our recent study reported on the fabrication and characterization of a neural conduit produced by collagen and soy protein isolate (SPI) for neural repair. We showed that the conduit can potentially act as a bridge to conduct neural regeneration [7]. The collagen-SPI conduits reduced the proliferation of human adult and fetal astrocyte and HMC3 cells, and decreased the gene expression of a few chemokines in adult human astrocytes, compared with collagen conduits in an in vitro analysis. We also found that collagen-SPI scaffolds reduced the gene expression of major histocompatibility complex class II (MHCII) molecules. This outcome indicates that the scaffolds can modulate the inflammatory reaction after neural injury [8].

Nanofiber scaffolds have shown promising applications in neural tissue regeneration [9,10]. Studies have reported that neural cell growth can be guided by the nanofibers [11–14]. One study showed that the aligned nanofibers directed neural stem cell adhesion and neurite elongation. The nanofibers functionalized with arginylglycylaspartic acid (RGD)-containing peptide GRGDSP polycaprolactone-RGD (PCL-RGD) promoted cell adhesion and proliferation [11]. Because their structure and diameter closely resemble the axonal morphology, nanofiber scaffolds can be grafted in injured spinal cord and peripheral nerve to support axonal regeneration.

Astrocytes participate in the neural repair process in the lesion by cleaning the damaged tissue, generating growth factors, and maintaining a permeable environment for axonal regrowth. However, the reactive astrocytes can also form a glial scar that blocks axonal growth. It was reported that after neural injury, fetal astrocytes, different from adult astrocytes, can potentially create a more conducive environment for neural regeneration [15–17]. Additionally, fetal astrocytes may produce factors for neuron protection and therefore promote neuron survival. Nanofibers made with SPI and collagen may regulate the immune response of neural tissue and act as a matrix carrier for the transplantation of cells such as fetal astrocytes. As human fetal astrocytes showed dynamic migration in an in vitro investigation, it is a good cell type for the study of cell-bioscaffold interaction. In this study, we report on the fabrication of nanofiber by SPI, collagen, and polycaprolactone (PCL). PCL is a biocompatible material and its incorporation in nanofibers can improve their integrity and mechanical property. The astrocyte growth on the composite nanofibers was characterized, and the cell transcriptome was analyzed by RNA-sequencing.

## MATERIALS AND METHODS

### Nanofiber fabrication

In this study, PCL (molecular weight of 80,000, Sigma-Aldrich, St. Louis, MO), SPI (MP Biomedicals, LLC, Santa Ana, CA), and type I bovine collagen were used to fabricate PCL, SPI/PCL, collagen/PCL and collagen/SPI/PCL nanofibers by electrospinning. To generate the PCL fibers, PCL was dissolved in 1,1,1,3,3,3-Hexafluoro-2-propanol (Oakwood Products, Inc., Estill, SC) in a concentration of 6% (w/v). To generate the composite fibers, SPI (2%)/PCL (4%), collagen (3%)/PCL (4%), collagen (1%)/SPI (1%)/PCL (4%) were dissolved in 1,1,1,3,3,3-Hexafluoro-2-propanol to form solutions for electrospinning. Fibers were collected on aluminum foil that covered a mandrel placed 15 cm away from the needle tip of an infusion syringe with a flow rate of 0.48 ml/hr. The applied voltage for electrospinning was 15 kV. To generate the random fibers, the mandrel rotation speed was set at 100 RPM, while the rotation speed was increased to 1500 RPM to fabricate the aligned fibers.

### Fiber characterization by FTIR assay, and contact angle assay

Fourier transform infrared spectroscopy was performed to study the nanofiber component. Spectra were obtained using a PerkinElmer Spectrum 3 FT-IR Spectrometer (PerkinElmer, Waltham, MA), which is equipped with a crystal plate that combines a diamond and zinc selenide (ZnSe) element designed for use in attenuated total reflectance (ATR) spectroscopy. To obtain an infrared (IR) spectrum, the specimen was first loaded onto the ATR sample platform. A swivel press was used to ensure the sample had optimal contact with the ATR crystal. IR light was then directed to the crystal where it interacted with the sample. During this process, some of the IR is absorbed by the sample, and the remaining light is reflected back to the detector, thus creating an IR spectrum.

The contact angles of deionized water drops on nanofiber membranes were measured by a goniometer (ramé-hart instrument co., Succasunna, NJ) at room temperature, and water drop pictures were taken. The procedure was repeated on three samples of each type of nanofiber, and the mean contact angle was calculated.

### Fetal astrocyte growth on nanofibers

The fetal human astrocytes (ScienCell Research Laboratories, Carlsbad, CA) were cultured with fetal astrocyte medium (ScienCell Research Laboratories, Carlsbad, CA) in an incubator (37°C) with 5% CO_2_. The nanofiber membrane on aluminum foil was cut as a round sample that fitted the 24-well plate or six-well plate wells.

### LIVE/DEAD^®^ cell viability assay

After the cells were cultured on nanofibers for two days, the cell viability was tested by LIVE/DEAD^®^ cell vitality kit (Life Technologies Corp., Grand Island, NY). The staining cell solution was made by adding EthD-1 stock solution (2 μl of 2 mM) and calcein AM stock solution (2 μl of 1 mM) into a cell culture medium (1 ml). The cells were incubated with the staining solution for 25 minutes at 37°C incubation before images were taken.

### SEM assay for nanofibers and cell-nanofiber interaction

After the fetal astrocytes (10,000 cells/nanofiber sample) were grown on the nanofiber for two days, they were washed with phosphate buffered saline (PBS) and fixed with 4% paraformaldehyde. The graded ethanol was used to dehydrate the fixed samples and further dried with hexamethyldisilazane. The dried samples were coated with gold for SEM imaging. A scanning electron microscope (JSM-6460LV, JEOL Inc., Peabody, MA) was utilized to take images of the cells and the nanofibers. The fiber diameter of SEM images was analyzed by National Institutes of Health (NIH) ImageJ software (Bethesda, MD). The nanofibers without cell seeding were coated with gold and studied by SEM. The fiber diameter for each type of nanofibers was analyzed by NIH ImageJ software.

### Immunocytochemistry

The cells grown on different types of fibers were fixed with 4% paraformaldehyde. The cells were permeabilized with 0.5% Triton X-100 for 15 minutes and blocked with 10% FBS for 30 minutes. All the cells were dual-labeled with anti-GFAP (glial fibrillary acidic protein) and anti-S100B antibodies. The cells were incubated with monoclonal anti-GFAP antibody (BioLegend, San Diego, CA) and secondary Alexa Fluor^®^ 594 donkey anti-mouse IgG (Jackson ImmunoResearch Laboratories, Inc., West Grove, PA) antibody. The cells were then incubated with polyclonal anti-S100B antibody (BioLegend, San Diego, CA) and secondary Alexa Fluor^®^ 488 goat anti-rabbit IgG (Jackson ImmunoResearch Laboratories, Inc., West Grove, PA) antibody. Fluorescent images were taken under the same exposure time using a florescent microscope (Zeiss Axio Observer). Four replications of each type of fibers were performed in this study. The fluorescent intensity of both red (GFAP) and green (S100B) fluorescence for the labeled cells was measured by NIH ImageJ and quantified. The ratio of fluorescent intensity of green and red fluorescence based on individual cells was quantified to present the differential expression of the two markers.

### Cell cycle study

The fetal astrocytes (150,000/nanofiber sample) were seeded in nanofiber samples fitting the six-well plate wells. After culturing for three days, the cells were collected by trypsin treatment and centrifugation. The cells were then fixed with cold (4°C) ethanol (70%) for at least one hour. The samples were centrifuged, and the supernatant was decanted. The cells were washed with PBS and centrifuged to remove supernatant. The FxCycle^™^ propidium iodide ribonuclease (PI/RNase) staining solution (0.5 ml) was used to stain the cells of each sample for 30 minutes at room temperature. The cells were analyzed by a CytoFLEX flow cytometer (Beckman Coulter, Brea, CA). The flowcytometry data analysis was performed by FlowJo^™^ Software (FlowJo LLC, Ashland, OR).

### Cell migration analysis

The fetal astrocytes were grown on the nanofiber samples (10,000 cells/sample) for two days. The cells were then labeled with Invitrogen^™^ wheat germ agglutinin (WGA) with Alexa Fluor^®^ 488 conjugation (5 μg/ml, Fisher Scientific International, Inc., Pittsburgh, PA) in cell culture medium for about one hour. The cell migration on nanofibers was recorded by a florescent microscope (Zeiss Axio Observer) covered by a plastic incubator with a temperature control (37°C) and CO_2_ supply. Sequential fluorescent images were captured every five minutes for a continuous three hours. All studies were repeated at least three times. The cell migration was tracked by NIH ImageJ software and analyzed by Chemotaxis and Migration Tool programs for cell migration velocity and distance.

### Next generation RNA gene sequencing assay

Fetal astrocytes (150,000/sample) were cultured on collagen/PCL or collagen/SPI/PCL nanofibers that fit in the six-well plate wells. After culturing for three days, the RNA was extracted from the cultured cells with a Direct-zol^™^ RNA Microprep Kit (Zymo Research, Irvine, CA) for RNA sequencing assay. RNA extraction was performed from the cells grown on three samples of each type nanofibers.

The extracted RNA was analyzed by mRNA-Seq using the Illumina NovaSeq 6000 Sequencing System at the University of Kansas Medical Center Genome Sequencing Facility (Kansas City, KS). Total RNA (500 ng) was used to initiate the Universal Plus mRNA-seq with NuQuant stranded mRNA library preparation protocol (Tecan Genomics 0520-A01). A S1000 thermal cycler (Bio Rad) was used to process the total RNA fraction by oligo dT beads capture enrichment of mRNA, fragmentation of mRNA, reverse transcription into cDNA, end repair of cDNA, and ligation with the appropriate unique dual index (UDI) adaptors with strand selection and library amplification by polymerase chain reaction (PCR) (12 cycles). Library validation was performed using the D1000 ScreenTape Assay kit (Agilent Technologies 5067–5582) on the Agilent TapeStation 4200.

### Data analysis of the RNA gene sequencing assay

Sequencing reads were processed using the nfcore/rnaseq pipeline (v3.16.0 (doi: 10.5281/zenodo.1400710)[18] for quality control, alignment with STAR [19] and quantification with RSEM.[20] The merged gene counts were evaluated for differential expression using the rsem-run-ebseq function of standalone RSEM (v1.3.3). Visualizations of RNASeq data were produced in R (R Core Team, 2023, R: A Language and Environment for Statistical Computing. R Foundation for Statistical Computing, Vienna, Austria). Normalized counts from EBSeq were used to calculate principal components with the prcomp function and the principal components analysis (PCA) plot was created with ggplot2 (Wickham 2016, ggplot2: Elegant Graphics for Data Analysis. Springer-Verlag, New York). A volcano plot based on differential expression results was produced with the plot function. The Database for Annotation, Visualization, and Integrated Discovery (DAVID) was used to identify KEGG signaling pathways enriched among up- and downregulated DEGs. Heatmaps of DEGs associated with enriched pathways were created with the R pheatmap package (Kolde R. pheatmap: Pretty Heatmaps, R package version 1.0.12. 2020) using log2-transformed, normalized gene counts with Z-score scaling by gene. Samples and genes were hierarchically clustered based on Euclidean distance.

### Statistical analysis

The data of research outcomes are described by mean ± standard deviation. The data was statistically analyzed by a two-tailed Student’s t-test and ANOVA using an SPSS program. A *p*-value of 0.05 is considered to be statistically significant.

## RESULTS

### Characterization of electrospun nanofibers

**Figure 1.**
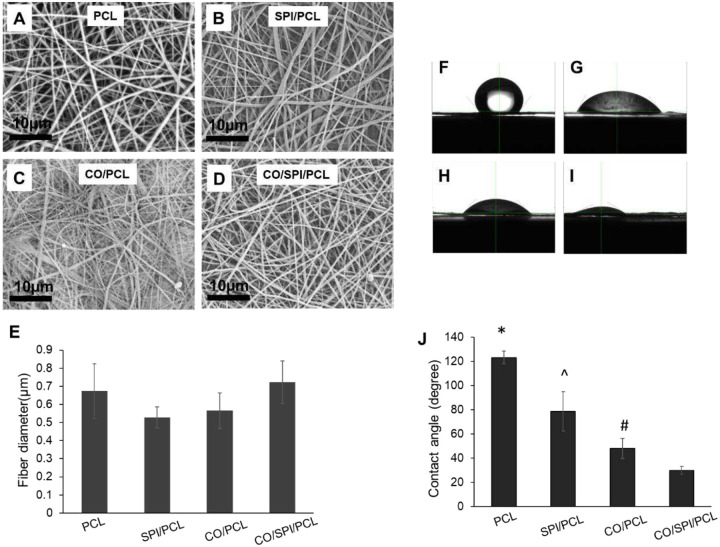
Fabricated nanofibers and measurement of contact angle. (A–D) SEM images of nanofibers: PCL (A), SPI/PCL (B), collagen/PCL (C), and collagen/SPI/PCL (D). (E) Quantification of nanofiber diameters. (F–I) Images showing contact angles of nanofibers: PCL (F), SPI/PCL (G), collagen (CO)/PCL (H), and collagen/SPI/PCL (I). (J) Quantification of nanofiber contact angle: *, compared with SPI/PCL, collagen/PCL and collagen/SPI/PCL nanofibers, p<0.05; ^^^, compared with SPI/PCL and collagen/SPI/PCL nanofibers, p<0.01; ^#^, compared with collagen/SPI/PCL nanofibers, p<0.01.

Four types of nanofibers (PCL, SPI/PCL, collagen (CO)/PCL, and CO/SPI/PCL) were generated by electrospinning (Figures 1A–D). The average fiber diameters of these four types of nanofibers are between 500 nm and 750 nm (Figure 1E). The contact angle of PCL fibers (132° ± 5.2°) is significantly higher than the collagen/PCL (78.7° ± 16.2°), SPI/PCL (48.0° ± 8.3°) and collagen/SPI/PCL fibers (29.9° ± 3.2°) (<0.05) (Figures F–J). The collagen and SPI in the nanofibers reduced the fiber contact angle significantly.

**Figure 2.**
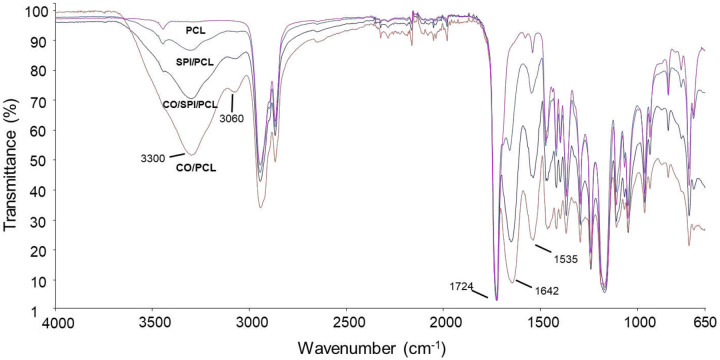
Normalized FTIR spectroscopy of nanofibers: PCL, SPI/PCL, collagen(CO)/PCL, and collagen/SPI/PCL nanofibers.

Fourier transform infrared (FTIR) spectroscopy was used to determine the functional group of type I collagen, SPI, and PCL in the nanofibers (Figure 2). This test shows the spectra obtained from FTIR for the different types of composite nanofibers. The intensified peaks at 1724 cm^−1^ indicates the presence of PCL in the nanofibers. Both collagen and SPI components in the nanofibers showed peaks at 1530–1550 cm^−1^ (N–H bending, amide II), and 1640–1660 cm^−1^ (C = O stretching, amide I). The collagen showed peaks at 3300 cm^−1^ (N–H bending, amide A), and 3060 cm^−1^ (C = O stretching, amide B).[21–23]

### Fetal astrocyte viability, and proliferation on nanofibers

**Figure 3.**
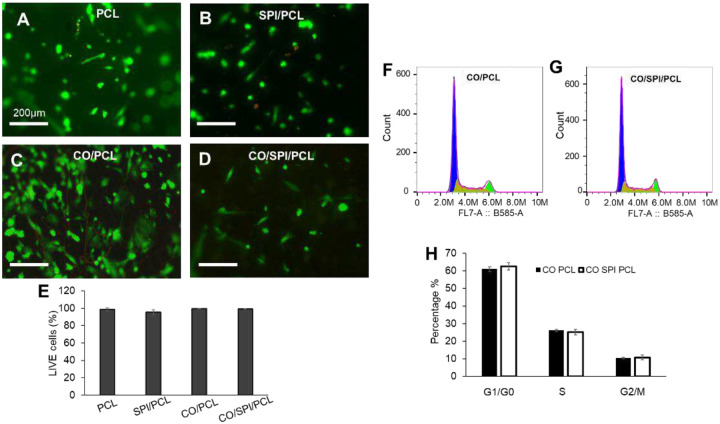
Cell viability and cell cycle assays for fetal astrocytes on nanofibers. (A–D) LIVE/DEAD^®^ assay shows live cells (green) and dead cells (red) on nanofibers: PCL (A), SPI/PCL (B), collagen/PCL (C), and collagen/SPI/PCL (D). Scale bar, 200 μm. (E) Quantification of ratio of live cells to total cells. (F–H) Flow cytometry assay of fetal astrocytes on nanofibers: DNA content histogram of astrocytes on CO/PCL (F), DNA content histogram of astrocytes on CO/SPI/PCL (G), and histogram of cell cycle distribution of astrocytes at G1/G0, S, and G2/M phases (H).

**Figure 4.**
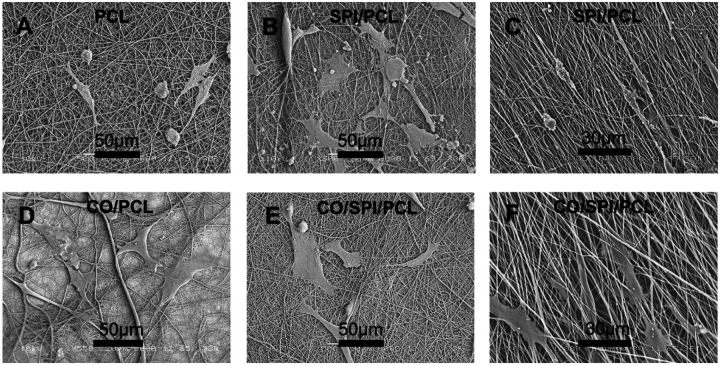
SEM images of fetal astrocytes on nanofibers matrices: (A) PCL random fiber, (B) SPI/PCL random fibers, (C) SPI/PCL aligned fibers, (D) collagen/PCL random fibers, (E) collagen/SPI/PCL random fibers, (F) collagen/SPI/PCL aligned fibers.

In this study, we investigated the fetal astrocyte viability on nanofibers using a LIVE/DEAD^®^ assay kit. Most cells grown on the nanofibers showed green fluorescence, indicating the live cells (Figures 3A–D). Quantification of the cells showed high cell viability on all types of nanofibers (Figure 3E). Flow cytometry was performed to study the cell cycle and proliferation on collagen/PCL and collagen/SPI/PCL fibers (Figures 3F, G). The cell at G1 phase showed a similar percentage of cell number for astrocytes grown on collagen/PCL (61.0% ± 1.3%) and collagen/SPI/PCL fibers (62.5% ± 2.1%) (Figure 3H). Scanning electron microscopy (SEM) images showed cell morphology on the nanofibers. The cells spread out and adhere to the random fibers (Figures 4A, B, D, E). The cells extend out protrusions to attach to the nanofibers. The cells on aligned fibers showed elongated cell morphology and orientation along the fibers (Figure 4C, F).

### The analysis of S100B and GFAP in fetal astrocyte by immunocytochemistry

**Figure 5.**
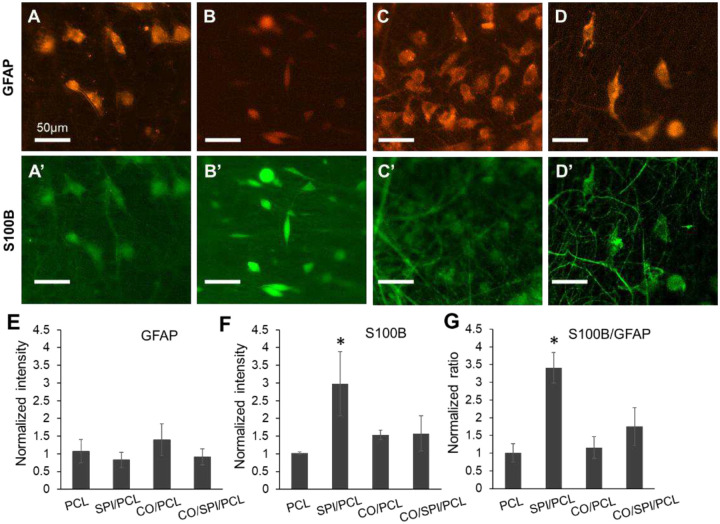
Immunostaining of S100B and GFAP for astrocytes grown in nanofibers. The cells were labeled with GFAP (A-D) and S100B (A’ to D’) antibodies on PCL (A, A’), SPI/PCL (B, B’), CO/PCL and CO/SPI/PCL nanofibers. The normalized fluorescent intensities show the expression of GFAP (C) and S100B (D) in astrocytes on nanofibers. (E) The normalized ratio of red to green fluorescent intensity shows the relative ratio of GFAP (red) versus S100B (green) in astrocytes on nanofibers. *, compared with PCL, CO/PCL, and CO/SPI/PCL nanofibers, p<0.01.

In this study, immunocytochemistry was performed for dual labeling of GFAP and S100B for fetal astrocytes on different fibers (Figure 5A–D). S100B and GFAP are specific markers of astrocyte. The study aims to study the expression of these markers on different types of nanofibers. The fluorescent intensity of the two markers in astrocytes was measured and quantified by ImageJ. The normalized S100B level in astrocytes on CO/SPI/PCL fibers was significantly higher than the cells on other types of fibers (p<0.05, Figure 5F), while the normalized GFAP level was not significantly different for cells on different types of fibers (Figure 5E). We further quantified the ratio of S100B to GFAP in cells on nanofibers. The S100B to GFAP ratio in cells on SPI/PCL was significantly higher than all the other groups (p<0.01, Figure 5G).

### Characterization of fetal astrocyte migration on nanofibers

**Figure 6.**
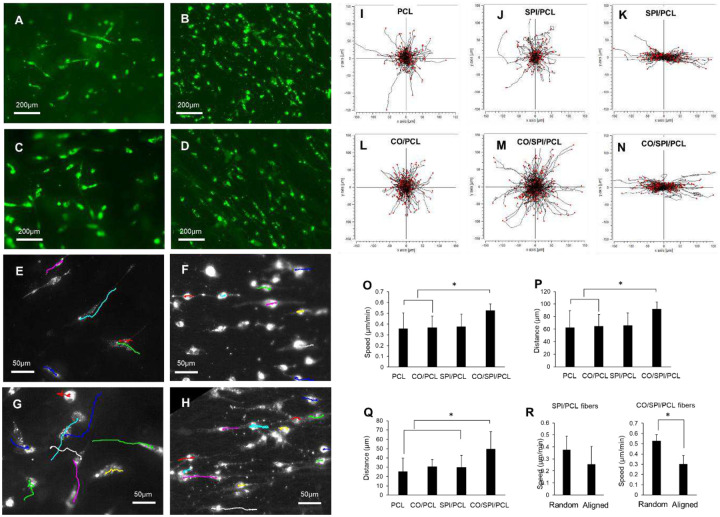
Analysis of astrocyte migration on nanofibers. (A, C) Growth of astrocytes on random nanofibers generated by SPI/PCL (A) and collagen/SPI/PCL (C). (B, D) Growth of astrocytes on aligned nanofibers generated by SPI/PCL (B) and collagen/SPI/PCL (D). (E, G) Tracking of astrocyte migration on aligned nanofibers generated by SPI/PCL (E) and collagen/SPI/PCL (G). (F, H) Tracking of astrocyte migration on aligned nanofibers generated by SPI/PCL (F) and collagen/SPI/PCL (H). Cells labeled with WGA with Alexa Fluor^®^ 488 conjugation. Scale bar, 200 μm. (I–N) Tracks of cell migration from at least three experiments, each line indicates one cell migration pathway, initial positions of all cells are in the center of each frame: Cell migration tracks on random fibers (I, J, L, M), and cell migration tracks on aligned collagen/SPI/PCL fibers (K, N). (O) Quantification of cell migration speed. (P) Quantification of accumulated cell migration distance. (Q) Quantification of Euclidean cell migration distance. (R) Comparison of cell migration speeds on random and aligned fibers. *, <0.05.

The cell growth and morphology on nanofibers were studied after the cells were labeled with Alexa Fluor^™^ 488 conjugated wheat germ agglutinin (WGA). These cells showed random orientation on random SPI/PCL (Figure 6A) and CO/SPI/PCL nanofibers (Figure 6C), while the cells showed oriented morphology on aligned SPI/PCL (Figure 6B) and CO/SPI/PCL nanofibers (Figure 6D). The cells migrated dynamically on both random nanofibers (SPI/PCL fibers, Figure 5E, see also supplemental material video 1; and CO/SPI/PCL fibers, Figure 6G, see also supplemental material video 2) and aligned nanofibers (SPI/PCL, Figure 6F, see also supplemental material video 3; and CO/SPI/PCL, Figure 6H, see also supplemental material video 4). The cell migration tracks are labeled with colored lines (Figures 6I–N). The cell migration speed on CO/SPI/PCL nanofibers (0.53 ± 0.06 μm/min) is significantly higher than that on CO/PCL (0.37 ± 0.11 μm/min) and PCL (0.36 ± 0.15 μm/min) nanofibers (Figure 6O). The accumulated and Euclidean distance of cell migration were quantified (Figure 6P). The Euclidean distance of cell migration on PCL and SPI/PCL nanofibers is significantly lower than that on CO/SPI/PCL fibers (Figure 6Q). The cells showed a significantly higher migration speed on CO/SPI/PCL random fibers compared with aligned fibers (Figure 6R). The migration speeds on aligned SPI/PCL and CO/SPI/PCL fibers are 0.25 ± 0.15 μm/min and 0.3 ± 0.08 μm/min respectively, while the migration speeds on random SPI/PCL and collagen/SPI/PCL fibers are 0.38 ± 0.11 μm/min and 0.53 ± 0.06 μm/min, respectively.

### Differential gene expression of astrocytes on nanofibers

**Figure 7.**
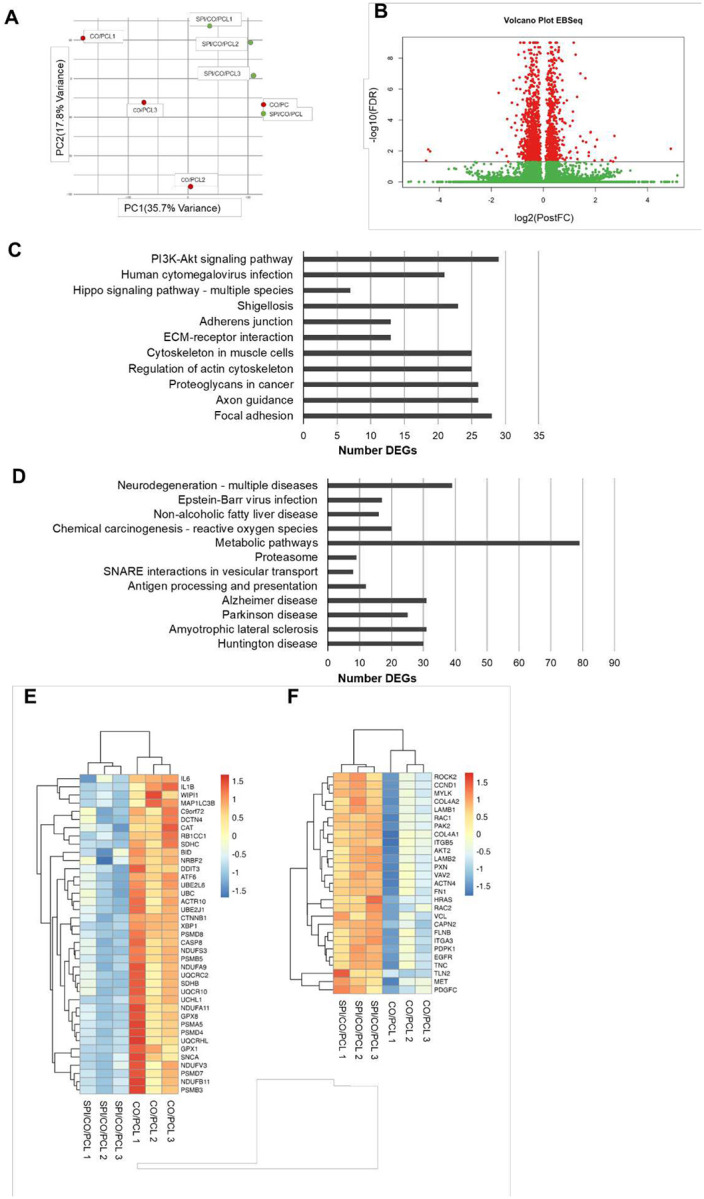
Analysis of RNA sequencing data. (A) Two-dimensional Principal Components Analysis (PCA) plot of RNA-seq samples based on normalized gene counts. Samples are color-coded by nanofiber type. (B) Volcano plot with log2(Posterior Fold Change) on the X axis and −log10(False Discovery Rate (FDR)) on the Y axis. Each point represents a gene, with red indicating genes that are significantly differentially expressed (FDR < 0.05) in fetal astrocytes on CO/SPI/PCL versus CO/PCL nanofibers and green denoting those that are not. Points to the right of the midline are up-regulated in cells on CO/SPI/PCL cells relative to cells on CO/PCL, while those to the left are down-regulated in cells on CO/SPI/PCL. (C) KEGG pathways enriched among up-regulated DEGs in astrocytes on CO/SPI/PCL nanofibers relative to cells on CO/PCL fibers. (D) KEGG pathways enriched among down-regulated DEGs in astrocytes on CO/SPI/PCL nanofibers relative to cells on CO/PCL fibers. Heatmaps of DEGs in the KEGG Neurodegeneration – multiple diseases (E) and Focal adhesion (F) pathways. The color scale represents the Z-score calculated across each gene. Genes (rows) and samples (columns) are clustered by Euclidean distance.

To compare gene expression in astrocytes grown on collagen/SPI/PCL nanofibers versus those grown on collagen/PCL nanofibers, we performed RNA-Seq on three samples from each condition. The total number of clean reads per RNA-Seq library ranged from 59.3 million to 66.5 million for cells on CO/PCL and from 55.2 million to 70.2 million for cells on CO/SPI/PCL. Over 95% of the reads from all samples mapped to the human genome (GRCh38), with over 85% aligning uniquely. Principal components analysis (PCA) of normalized gene expression showed a clear separation of samples from cells on CO/PCL and CO/SPI/PCL nanofibers (Figure 7A). Differential gene expression analysis identified 652 up-regulated and 835 down-regulated genes (p < 0.05) in astrocytes on CO/SPI/PCL nanofibers relative to those on CO/PCL nanofiber (Figure 7B).

### Analysis of differentially expressed genes

**Figure 8.**
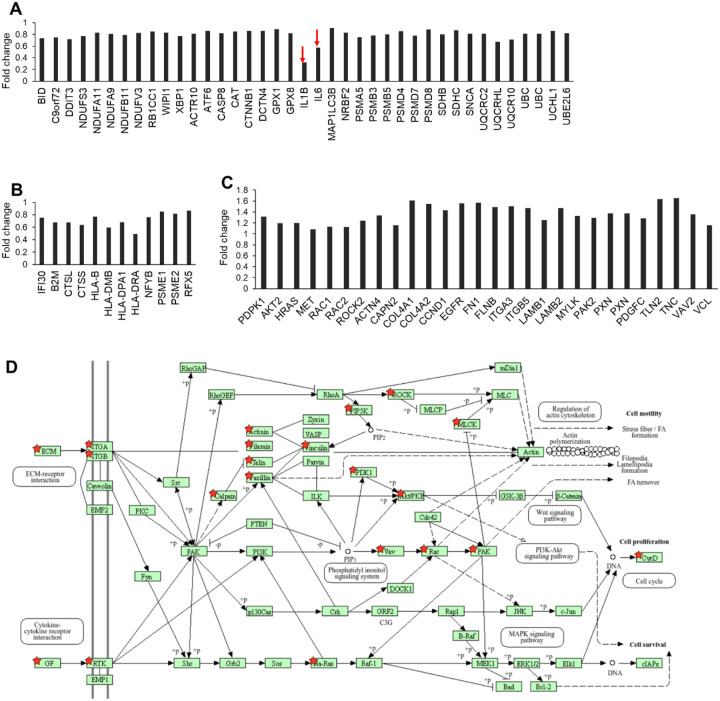
Fold changes of DEGs in enriched KEGG signaling pathways. DEGs in enriched KEGG signaling pathways of neurodegeneration (A), and antigen processing and presentation (B), which are significantly down-regulated in fetal astrocytes grown on collagen/SPI/PCL fibers compared with collagen/PCL. (C) DEGs in the enriched KEGG signaling pathway of focal adhesion, which are significantly up-regulated in fetal astrocytes grown on collagen/SPI/PCL fibers compared with collagen/PCL. (D) KEGG pathway of focal adhesion; red stars indicate up-regulated genes.

Up- and down-regulated DEGs were separately analyzed for enrichment of Kyoto Encyclopedia of Genes and Genomes (KEGG) pathways. Our results showed that 11 signaling pathways are enriched among up-regulated genes (Figure 7C), and 12 pathways are enriched among down-regulated genes (Figure 7D). The pathways enriched among down-regulated genes include a few pathways that regulate neurodegeneration such as Parkinson’s disease, Alzheimer disease, amyotrophic lateral sclerosis, and Huntington’s disease. The down-regulated DEGs (39 DEGs) summarized in the “neurodegeneration - multiple diseases” pathway are involved in the processes of these diseases (Figures 7E and 8A). In particular, the significant down-regulation of IL1B and IL6 genes by the astrocytes on collagen/SPI/PCL fibers compared with the cells on collagen/PCL fibers suggests regulation of the immune response by SPI. The down-regulated pathways also include “antigen processing and presentation” (12 DEGs) (Figure 8B). The DEGs in this pathway include four human leukocyte antigen (HLA) genes (HLA-B, HLA-DMB, HLA-DPA1, and HLA-DRA). These genes encode molecules that regulate CD4^+^ T cell function and therefore play a critical role in the immune reaction and autoimmune diseases. The “focal adhesion” pathway, which is enriched among up-regulated genes (28 DEGs), includes several genes (COL4A1, COL4A2, FN1, LAMB1, LAMB2, AKT2, RAC1, RAC2, ROCK2, PIP5K1A) that encode molecules likely to regulate astrocyte adhesion and motility on collagen/SPI/PCL substrate (Figures 7F, 8C, and 8D).

## DISCUSSION

Biomaterial neural conduits have been reported in numerous studies for spinal cord and peripheral nerve repair [24–26]. The structural design of a neural bioscaffold is the research target to create a permissive microenvironment and improve axonal growth. Nanofibers have been considered an attractive type of scaffold for neural repair application because they mimic the structure of extracellular matrix structure and axonal morphology [27–29]. Soy protein has shown promising application in tissue repair [3,30,31]. However, its potential for neural regeneration has been rarely studied. In our previous study, we reported the fabrication of a SPI-collagen hybrid protein neural conduit. SPI biocompatibility and the effect of SPI on astrocyte proliferation have been reported in our previous study [32]. Fetal astrocytes showed a lower cell proliferation rate when they were grown in SPI/collagen hydrogel compared with collagen hydrogel, thereby indicating that the SPI component can reduce cell proliferation. In this study, we observed that the fetal astrocytes showed high cell viability on collagen/SPI/PCL nanofibers that contain SPI as collagen/PCL nanofibers. We also found that the G1, S, and M phases in the cell cycle of fetal astrocytes on collagen/PCL or collagen/SPI/PCL were not significantly different, indicating that the SPI component in the nanofiber does not affect the cell cycle. Cyclin A regulates DNA replication at S-phase and mitosis. Cyclin B1 and B2 activate the cell transition from G2 phase to mitosis [33]. Cyclin D1 controls the cell transition from the G1 phase to the S phase in a cell cycle[34]. The RNA sequencing data showed that the folds for the gene expression of CCNB1, CCNB2, and CCNA2 are 1.25, 1.17, and 1.08 respectively for the cells on CO/PCL versus CO/SPI/PCL fibers. However, the fold for the gene expression of CCND1 is 0.68 for the cells on CO/PCL versus CO/SPI/PCL fibers. The combined function of these gene expressions may explain that the cells do not show significant difference at different phases in their cell cycles on CO/PCL and CO/SPI/PCL nanofibers.

Although both GFAP and S100B are astrocyte markers, they express at different stages of neural development [35]. GFAP is expressed at early stage in neural development, while S100B is expressed at more mature stage of astrocytic development state. In this study, we observed that the fluorescent intensities of S100B and GFAP are not significantly different between CO/PCL and CO/SPI/PCL groups. The finding is consistent with the RNA sequencing data that showed that the gene expression of S100B and GFAP was not significantly different between CO/PCL and CO/SPI/PCL groups. It is interesting to observe the higher S100B level in astrocytes grown on SPI/PCL nanofiber compared with other nanofibers, while the GFAP level was not significantly different among those groups. Further study is needed to explore the function of soy protein on fetal astrocyte maturation.

Cell migration is an important cellular activity in the tissue regeneration process [36–39]. Cell migration has been observed on nanofibers, and the topography of nanofiber matrices can significantly affect cell morphology and migration [40,41]. In this study, we observed the effect of material component in nanofibers on fetal astrocyte migration. The fetal astrocyte motility is an important cellular behavior because it represents the capability of cell distribution within the lesion after they are grafted. In a previous study, we found that the fetal astrocytes migrated dynamically on SPI/collagen at a migration speed of 0.64 ± 0.08 μm/min, and collagen substrates at a migration speed of 0.61 ± 0.07 μm/min. The cell migration velocities were not significantly different on those matrices. In this study, we found that the cell migration velocity on CO/SPI/PCL fibers is significantly higher than that on CO/PCL fibers.

A number of studies have reported that the aligned nanofibers can guide the orientation of cell growth [9,42,43]. It was also reported that the axon migration was guided by the fibers [44]. As shown in these studies, we observed the fetal astrocyte orientation along the aligned fibers. However, the migration of the astrocyte-aligned nanofibers has rarely been demonstrated. It is not clear if the nanofiber can guide the cell body movement. In this study, we compared cell migration on random and aligned nanofibers. We found the aligned nanofibers directed the cell orientation of adhesion and guided the cell migration. The oriented migration may allow more effective cell translocation between the lesion and healthy neural tissue. Interestingly, the migration velocities of fetal astrocytes on aligned SPI/PCL and collagen/SPI/PCL nanofibers fibers were significantly lower than the cells on random fibers. The aligned fiber that restricted the direction of migration may slow down the cell migration speed.

In this study, we observed that up-regulated genes in cells grown on CO/SPI/PCL nanofibers were enriched in focal adhesion pathway genes that regulate cell adhesion and migration. Inclusion of the SPI component resulted in higher gene expression of extracellular matrix (ECM) proteins including collagen type IV alpha 1 chain (COL4A1) and collagen type IV alpha 2 chain (COL4A2), fibronectin 1(FN1), laminin subunit beta 1(LAMB1), and laminin subunit beta 2(LAMB2). These ECM proteins are critical molecules that mediate cell adhesion and migration. Collagen can be recognized by cell integrin receptors and provide structural support for cell attachment. Fibronectin has a collagen-binding site and can organize the ECM components. Fetal astrocytes also increased the integrin subunit gene expression including integrin subunit alpha 3 (ITGA3), beta 5 (ITGB5), beta 1 (LAMB1), and beta 2 (LAMB2). The cell surface receptors are crucial in the cell-ECM interaction. The increased expression of these genes stimulates cell migration. Both focal adhesion and the PI3K-AKT signaling pathway are enriched in up-regulated genes. The up-regulated genes in both pathways include AKT serine/threonine kinase 2(AKT2) [45], Rac family small GTPase 1(RAC1), Rac family small GTPase 2(RAC2),[46] Rho-associated coiled-coil-containing protein kinase 2(ROCK2) [47], and phosphatidylinositol-4-phosphate 5-kinase type 1 alpha (PIP5K1A). These molecules are involved in the regulation of cell morphology and migration. AKT and PIP5K are important for cell survival. Phosphatidylinositol 4-phosphate 5-kinase (PIP2) generated by PIP5K is involved in cell membrane dynamics and cell motility. AKT is a key molecule regulating cell growth and inhibition of cell apoptosis. Rock2, referred to as Rho-associated protein kinase, and Rac2, a small GTPase protein, are key molecules that regulate cytoskeleton organization, which is essential for cell motility, cell morphology, and cell adhesion. In this study, we observed the up-regulation of those genes, suggesting that the SPI component in nanofibers promoted cell adhesion, cell morphological development, and cell migration. We also observed the higher migration distance for fetal astrocytes on CO/SPI/PCL nanofibers compared with CO/PCL fibers. The higher gene expression of the above molecules may explain the higher cell migration speed.

KEGG pathways that are enriched among down-regulated genes are “neurodegeneration-multiple diseases” and “antigen processing and presentation.” In particular, the down-regulation of IL1B and IL6 genes helps to reduce the inflammatory reaction. The down-regulation of major histocompatibility complex genes HLA-B, HLA-DMB, HLA-DPA1, and HLA-DRA will reduce the activation of CD4^+^ T cells [48,49]. In a previous study, we studied the gene expression of human microglia on collagen or collagen/SPI substrates by RNA-Seq. We reported that among down-regulated genes, the “antigen processing and presentation” pathway showed enrichment, primarily due to the down-regulation of MHCII molecules [8]. These studies suggest that SPI may regulate the immune response by reducing the HLA gene expression in fetal astrocytes and microglia.

## Supplementary Material

Supplementary Files

This is a list of supplementary files associated with this preprint. Click to download.

- supplementalmaterialvideo1.avi
- supplementalmaterialvideo2.avi
- supplementalmaterialvideo3.avi
- supplementalmaterialvideo4.avi

supplemental material video 1, fetal astrocyte migration on random SPI/PCL fibers.

supplemental material video 2, fetal astrocyte migration on random SPI/CO/PCL fibers.

supplemental material video 3, fetal astrocyte migration on aligned SPI/PCL fibers.

supplemental material video 4, fetal astrocyte migration on aligned SPI/CO/PCL fibers.

## Data Availability

All data generated or analysed during this study are included in this published article.
